# The genome sequence of the Kent black arches, *Meganola albula* (Denis & Schiffermüller, 1775)

**DOI:** 10.12688/wellcomeopenres.18672.1

**Published:** 2022-12-22

**Authors:** Douglas Boyes, Ian Sims, David Lees, University of Oxford and Wytham Woods Genome Acquisition Lab, Natural History Museum Genome Acquisition Lab, Darwin Tree of Life Barcoding collective, Wellcome Sanger Institute Tree of Life programme, Wellcome Sanger Institute Scientific Operations: DNA Pipelines collective, Tree of Life Core Informatics collective, Alexander Dove, Darwin Tree of Life Consortium

**Affiliations:** 1 UK Centre for Ecology and Hydrology, Wallingford, Oxfordshire, UK; 2 British Entomological and Natural History Society, Reading, Berkshire, UK; 3 Natural History Museum, London, UK; 4 Wellcome Sanger Institute, Hinxton, UK

**Keywords:** Meganola albula, Kent Black Arches, genome sequence, chromosomal, Lepidoptera

## Abstract

We present a genome assembly from an individual male *Meganola albula* (the Kent black arches; Arthropoda; Insecta; Lepidoptera; Nolidae). The genome sequence is 405 megabases in span. Most of the assembly (99.95%) is scaffolded into 30 chromosomal pseudomolecules with the Z sex chromosome assembled. The mitochondrial genome has also been assembled and is 15.4 kilobases in length.

## Species taxonomy

Eukaryota; Metazoa; Ecdysozoa; Arthropoda; Hexapoda; Insecta; Pterygota; Neoptera; Endopterygota; Lepidoptera; Glossata; Ditrysia; Noctuoidea; Nolidae; Nolinae; *Meganola*; *Meganola albula* (Denis & Schiffermüller, 1775) (NCBI:txid987977).

## Background

Kent Black Arches, or *Meganola albula*, belonging to the moth family *Nolidae* ( Zahiri *et al.*, 2013) and one of the 80 species in the genus *Meganola* ( Cha *et al.*, 2019), was first described in 1775 by Denis & Schiffermüller in Austria. The species bears close similarity to Scarce Black Arches ( *Nola aerugula*), another species found in similar coastal UK habitats as Kent Black Arches ( NBN Atlas; Õunap *et al.*, 2021). *M. albula* wings have a white ground colour with a curved brown medial band, white curved sub-terminal line and brown terminal field ^.^( Perveen & Khan, 2017). The *M. albula* wingspan is 16–20 mm ( Cha *et al.*, 2019; Kiss, 2017; Zahiri *et al.*, 2012). The head and thorax are white, and the abdomen is brown ( Cha *et al.*, 2019).

This species is widely distributed across the Palearctic region ( Tshistjakov, 2008); most observations have been reported across Western Europe, seen as far north as the southern coast of Finland. *M. albula* is sparsely distributed across the UK, primarily found in coastal regions. Early sightings within the UK were along the southern coast of England ( Heath & Alford, 1973), primarily within counties of Kent and Sussex, but the species is more frequently seen further north along the south-east coastline ( NBN Atlas). *M. albula* occupies a variety of coastal habitats, including coastal marshes ( Zilli, 2014) and woodlands, with the larva feeding on *Rubus caesius* ( Seymour, 2018), *Fragaria* (strawberry), *Potentilla*, *Vaccinium*, *Lotus*, *Trifolium* and *Mentha* ( Tshistjakov, 2008).

The adult *M. albula* is most commonly seen within the UK and western Europe between June and August, with most sightings occurring during the month of July ( NBN Atlas), flying in a single generation. In eastern regions (far eastern Russian, eastern China) multiple flight generations are seen, from towards the end of May till June and then from July into early August ( Tshistjakov, 2008). This species overwinters as small larvae ( Skinner & Wilson, 2009).

## Genome sequence report

**Figure 1.**
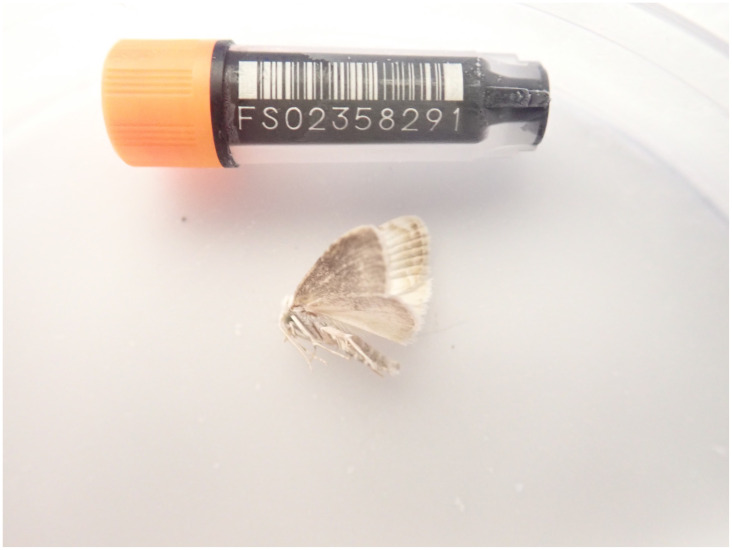
The *Meganola albula* (ilMegAlbu1) specimen used for genome sequencing.

The genome was sequenced from one male *M. albula* specimen ( Figure 1) collected from Wytham Woods, Berkshire, UK (latitude 51.769, longitude –1.33). A total of 43-fold coverage in Pacific Biosciences single-molecule HiFi long reads and 108-fold coverage in 10X Genomics read clouds was generated. Primary assembly contigs were scaffolded with chromosome conformation Hi-C data. Manual assembly curation corrected 10 missing or misjoins, reducing the scaffold number by 15%.

**Figure 2.**
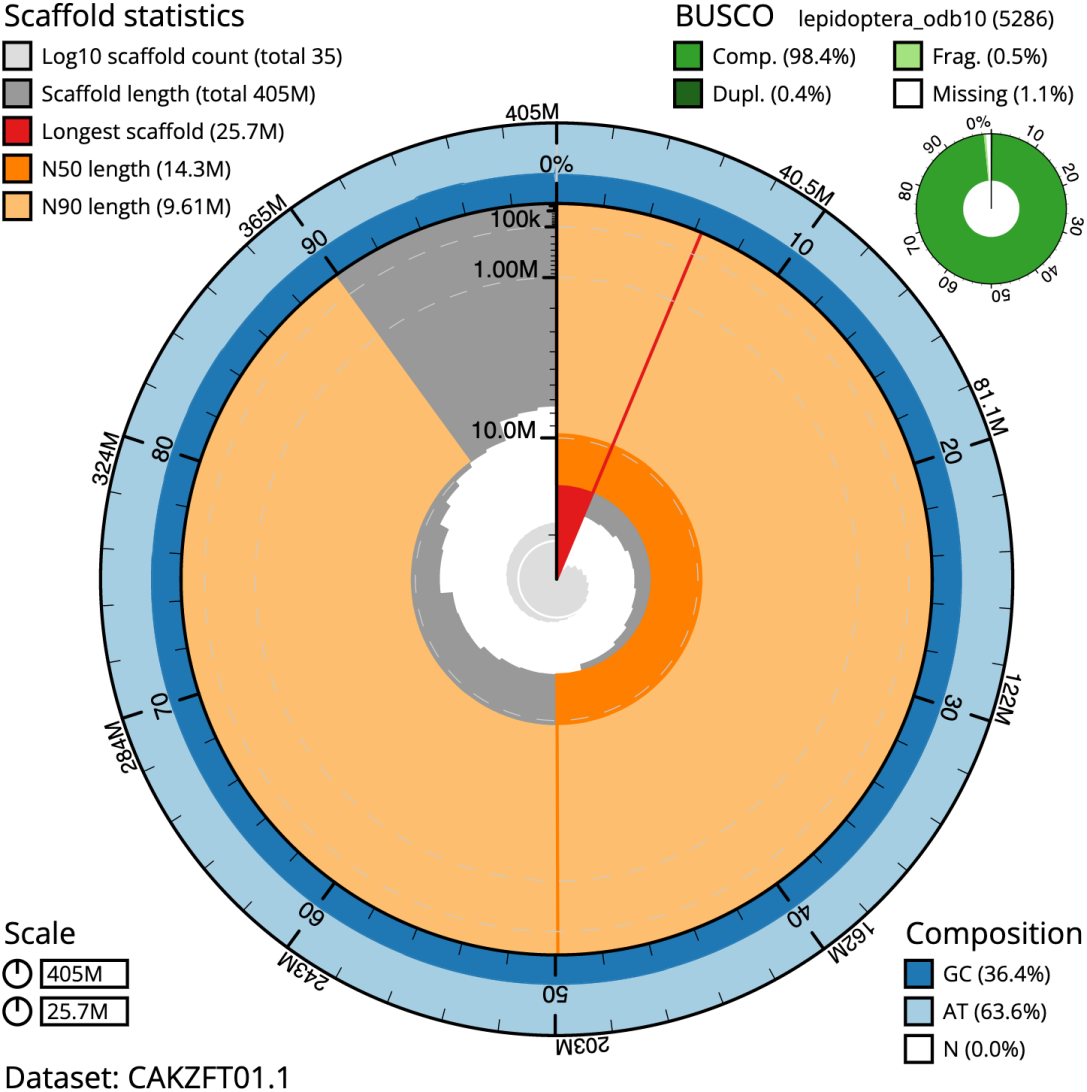
Genome assembly of *Meganola albula*, ilMegAlbu1.1: metrics. The BlobToolKit Snailplot shows N50 metrics and BUSCO gene completeness. The main plot is divided into 1,000 size-ordered bins around the circumference with each bin representing 0.1% of the 405,261,716 bp assembly. The distribution of chromosome lengths is shown in dark grey with the plot radius scaled to the longest chromosome present in the assembly (25,687,960 bp, shown in red). Orange and pale-orange arcs show the N50 and N90 chromosome lengths (14,254,146 and 9,608,783 bp), respectively. The pale grey spiral shows the cumulative chromosome count on a log scale with white scale lines showing successive orders of magnitude. The blue and pale-blue area around the outside of the plot shows the distribution of GC, AT and N percentages in the same bins as the inner plot. A summary of complete, fragmented, duplicated and missing BUSCO genes in the lepidoptera_odb10 set is shown in the top right. An interactive version of this figure is available at https://blobtoolkit.genomehubs.org/view/ilMegAlbu1.1/dataset/CAKZFT01.1/snail.

The final assembly has a total length of 405.2 Mb in 34 sequence scaffolds with a scaffold N50 of 14.3 Mb ( Table 1). Most (99.95%) of the assembly sequence was assigned to 30 chromosomal-level scaffolds, representing 29 autosomes and the Z sex chromosome ( Figure 2– Figure 5; Table 2). Chromosome-scale scaffolds confirmed by the Hi-C data are named in order of size. While not fully phased, the assembly deposited is of one haplotype. Contigs corresponding to the second haplotype have also been deposited. The assembly has a BUSCO v5.3.2 ( Manni *et al.*, 2021) completeness of 98.4% (single 98.1%, duplicated 0.4%) using the lepidoptera_odb10 reference set.

**Table 1.** Genome data for *Meganola albula*, ilMegAlbu1.1.

Project accession data	
Assembly identifier	ilMegAlbu1.1
Species	Meganola albula
Specimen	ilMegAlbu1
NCBI taxonomy ID	987977
BioProject	PRJEB46332
BioSample ID	SAMEA7701294
Isolate information	ilMegAlbu1: male, whole organism (genome assembly); ilMegAlbu2: unknown sex, (Hi-C); ilMegAlbu4: head/thorax (RNA-Seq)
Assembly metrics *	
Consensus quality (QV)	60.6 (Benchmark: ≥50)
*k*-mer completeness	100% (Benchmark: ≥95%)
BUSCO **	C:98.4%[S:98.1%,D:0.4%],F:0.5%,M:1.1%,n:5,286 (Benchmark: C ≥ 95%)
Percentage of assembly mapped to chromosomes	99.95% (Benchmark: ≥ 95%)
Sex chromosomes	ZZ (Benchmark: localised homologous pairs)
Organelles	Mitochondrial genome 15.4 kb (Benchmark: complete single alleles)
Raw data accessions	
PacificBiosciences SEQUEL II	ERR6808014
10X Genomics Illumina	ERR6688590–ERR6688593
Hi-C Illumina	ERR6688594
PolyA RNA-Seq Illumina	ERR10123654
Genome assembly	
Assembly accession	GCA_936450015.1
Accession of alternate haplotype	GCA_936440415.1
Span (Mb)	405.2
Number of contigs	47
Contig N50 length (Mb)	13.3
Number of scaffolds	34
Scaffold N50 length (Mb)	14.3
Longest scaffold (Mb)	25.7

* Assembly metric benchmarks are adapted from column VGP-2020 of “ Table 1: Proposed standards and metrics for defining genome assembly quality” from ( Rhie *et al.*, 2021). ** BUSCO scores based on the lepidoptera_odb10 BUSCO set using v5.3.2. C = complete [S = single copy, D = duplicated], F = fragmented, M = missing, n = number of orthologues in comparison. A full set of BUSCO scores is available at https://blobtoolkit.genomehubs.org/view/ilMegAlbu1.1/dataset/CAKZFT01.1/busco.

**Figure 3.**
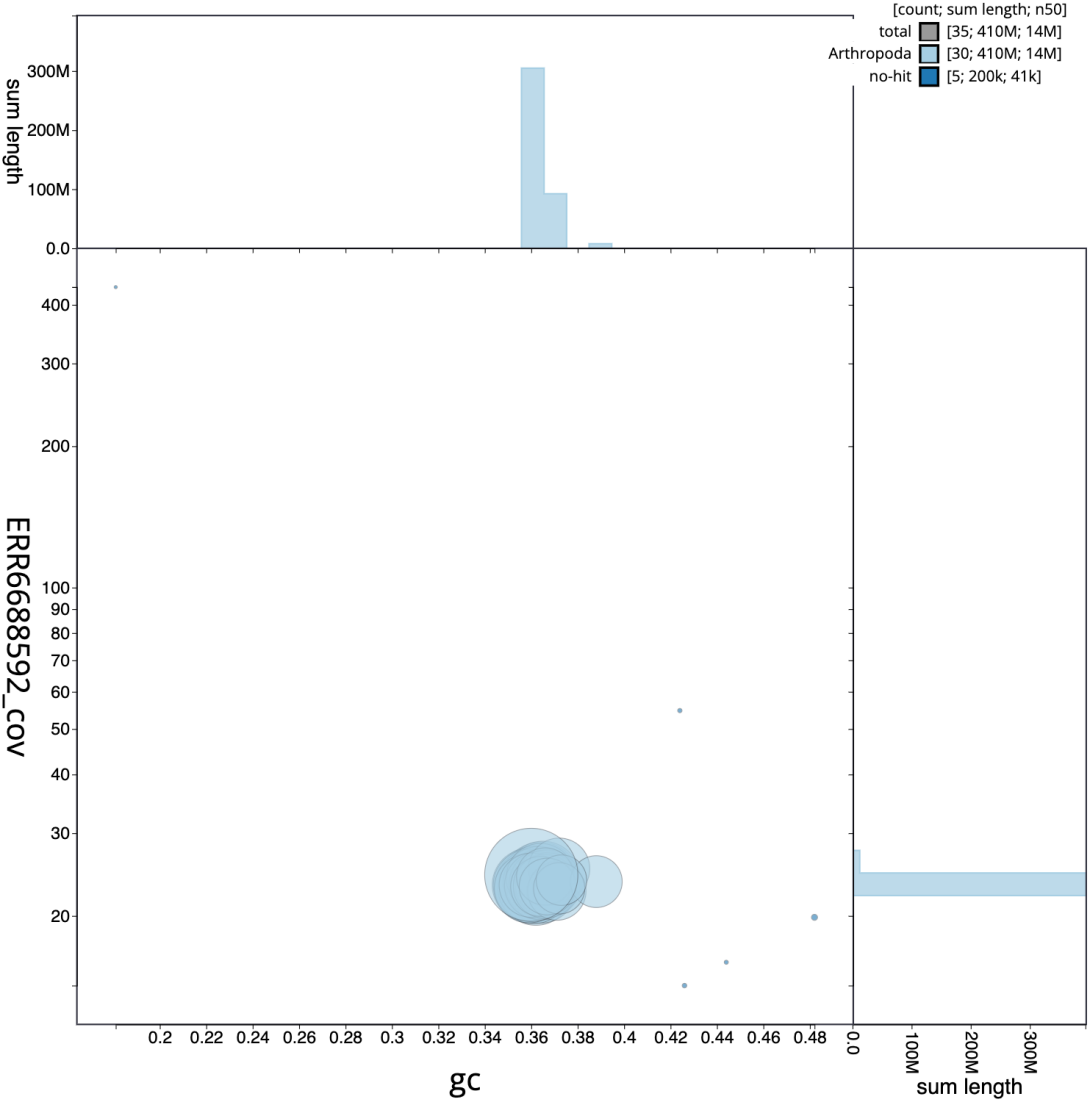
Genome assembly of *Meganola albula*, ilMegAlbu1.1: GC coverage. BlobToolKit GC-coverage plot. Chromosomes are coloured by phylum. Circles are sized in proportion to chromosome length. Histograms show the distribution of chromosome length sum along each axis. An interactive version of this figure is available at https://blobtoolkit.genomehubs.org/view/ilMegAlbu1.1/dataset/CAKZFT01.1/blob.

**Figure 4.**
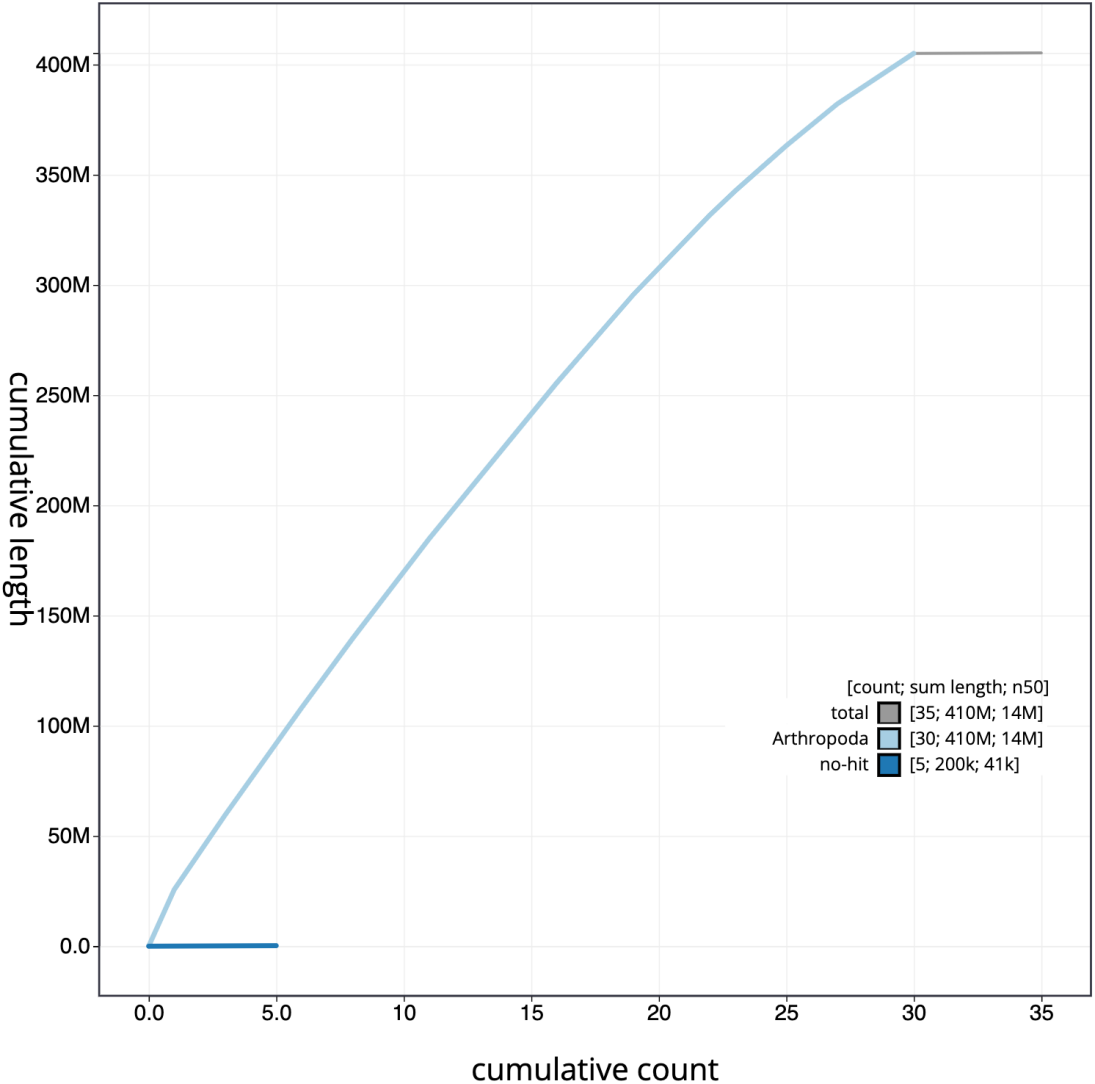
Genome assembly of *Meganola albula*, ilMegAlbu1.1: cumulative sequence. BlobToolKit cumulative sequence plot. The grey line shows cumulative length for all chromosomes. Coloured lines show cumulative lengths of chromosomes assigned to each phylum using the buscogenes taxrule. An interactive version of this figure is available at https://blobtoolkit.genomehubs.org/view/ilMegAlbu1.1/dataset/CAKZFT01.1/cumulative.

**Figure 5.**
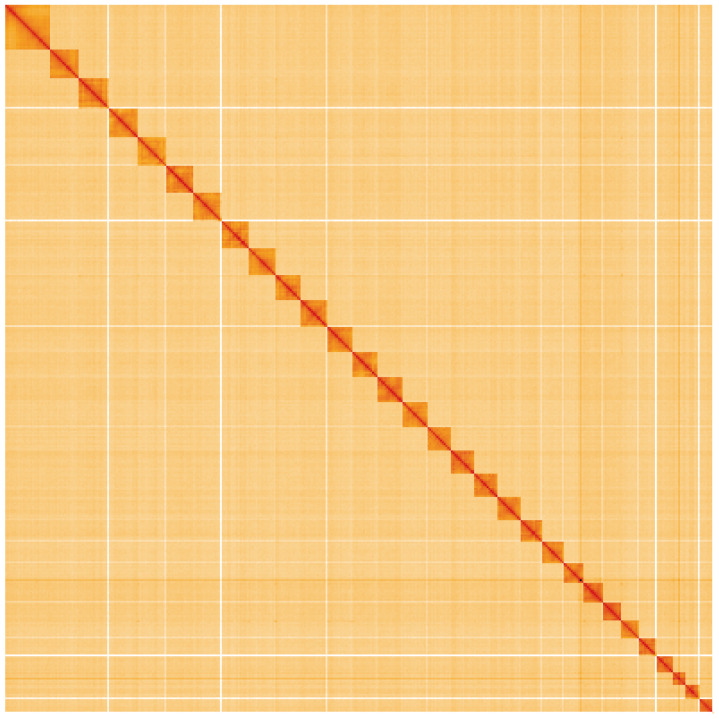
Genome assembly of *Meganola albula*, ilMegAlbu1.1: Hi-C contact map. Hi-C contact map of the ilMegAlbu1.1 assembly, visualised using HiGlass. Chromosomes are shown in order of size from left to right and top to bottom. An interactive version of this figure may be viewed at https://genome-note-higlass.tol.sanger.ac.uk/l/?d=G0f3dXHkQLiKTZCRbaZhOQ.

**Table 2.** Chromosomal pseudomolecules in the genome assembly of *Meganola albula*, ilMegAlbu1.

INSDC accession	Chromosome	Size (Mb)	GC%
OW388143.1	1	17.05	36.1
OW388144.1	2	16.89	36.4
OW388145.1	3	16.48	36.5
OW388146.1	4	16.13	36.2
OW388147.1	5	16.03	36.2
OW388148.1	6	15.8	35.9
OW388149.1	7	15.62	36
OW388150.1	8	15.28	36.4
OW388151.1	9	14.97	36.1
OW388152.1	10	14.96	35.9
OW388153.1	11	14.26	36.4
OW388154.1	12	14.25	36.4
OW388155.1	13	14.23	36.3
OW388156.1	14	14.13	35.9
OW388157.1	15	13.83	36.4
OW388158.1	16	13.39	36.5
OW388159.1	17	13.35	36.3
OW388160.1	18	13.25	36.7
OW388161.1	19	12.09	36.7
OW388162.1	20	12.09	36
OW388163.1	21	11.92	36.6
OW388164.1	22	10.99	37.2
OW388165.1	23	10.47	36.3
OW388166.1	24	9.99	37.1
OW388167.1	25	9.61	36.6
OW388168.1	26	9.4	36.7
OW388169.1	27	7.83	38.8
OW388170.1	28	7.64	37.2
OW388171.1	29	7.42	37.3
OW388142.1	Z	25.69	36
OW388172.1	MT	0.02	18.3

## Methods

### Sample acquisition and nucleic acid extraction

Two *M. albula* specimens (ilMegAlbu1 and ilMegAlbu2) of undetermined sex were collected using a light trap in Wytham Woods, Berkshire, UK (latitude 51.769, longitude –1.33) by Douglas Boyes (University of Oxford), who also identified the species. The specimens were identified by Douglas Boyes and snap-frozen on dry ice. A second specimen (ilMegAlbu4) of undetermined sex was collected using an aerial net from Hartslock Nature Reserve (latitude 51.51, longitude –1.11) by Ian Sims (British Entomological and Natural History Society). The specimen was identified by Ian Sims and David Lees (Natural History Museum, London) and snap-frozen on dry ice.

DNA was extracted at the Tree of Life laboratory, Wellcome Sanger Institute. The ilMegAlbu1 sample was weighed and dissected on dry ice. Whole organism tissue was disrupted using a Nippi Powermasher fitted with a BioMasher pestle. High molecular weight (HMW) DNA was extracted using the Qiagen MagAttract HMW DNA extraction kit. Low molecular weight DNA was removed from a 20 ng aliquot of extracted DNA using 0.8X AMpure XP purification kit prior to 10X Chromium sequencing; a minimum of 50 ng DNA was submitted for 10X sequencing. HMW DNA was sheared into an average fragment size of 12–20 kb in a Megaruptor 3 system with speed setting 30. Sheared DNA was purified by solid-phase reversible immobilisation using AMPure PB beads with a 1.8X ratio of beads to sample to remove the shorter fragments and concentrate the DNA sample. The concentration of the sheared and purified DNA was assessed using a Nanodrop spectrophotometer and Qubit Fluorometer and Qubit dsDNA High Sensitivity Assay kit. Fragment size distribution was evaluated by running the sample on the FemtoPulse system.

RNA was extracted from head/thorax tissue of ilMegAlbu4 in the Tree of Life Laboratory at the WSI using TRIzol, according to the manufacturer’s instructions. RNA was then eluted in 50 μl RNAse-free water and its concentration was assessed using a Nanodrop spectrophotometer and Qubit Fluorometer using the Qubit RNA Broad-Range (BR) Assay kit. Analysis of the integrity of the RNA was done using Agilent RNA 6000 Pico Kit and Eukaryotic Total RNA assay. 

### Sequencing

Pacific Biosciences HiFi circular consensus and 10X Genomics read cloud DNA sequencing libraries were constructed according to the manufacturers’ instructions. Poly(A) RNA-Seq libraries were constructed using the NEB Ultra II RNA Library Prep kit. DNA and RNA sequencing was performed by the Scientific Operations core at the WSI on Pacific Biosciences SEQUEL II (HiFi) and Illumina NovaSeq 6000 (RNA-Seq and 10X) instruments. Hi-C data were also generated from whole organism tissue of ilMegAlbu2 using the Arima v2 kit and sequenced on the Illumina NovaSeq 6000 instrument.

### Genome assembly

Assembly was carried out with Hifiasm ( Cheng *et al.*, 2021) and haplotypic duplication was identified and removed with purge_dups ( Guan *et al.*, 2020). One round of polishing was performed by aligning 10X Genomics read data to the assembly with Long Ranger ALIGN, calling variants with freebayes ( Garrison & Marth, 2012). The assembly was then scaffolded with Hi-C data ( Rao *et al.*, 2014) using SALSA2 ( Ghurye *et al.*, 2019). The assembly was checked for contamination as described previously ( Howe *et al.*, 2021). Manual curation was performed using HiGlass ( Kerpedjiev *et al.*, 2018) and Pretext ( Harry, 2022). The mitochondrial genome was assembled using MitoHiFi ( Uliano-Silva *et al.*, 2021), which performed annotation using MitoFinder ( Allio *et al.*, 2020). The genome was analysed and BUSCO scores generated within the BlobToolKit environment ( Challis *et al.*, 2020). Table 3 contains a list of all software tool versions used, where appropriate.

**Table 3.** Software tools and versions used.

Software tool	Version	Source
BlobToolKit	3.4.0	( Challis *et al.*, 2020)
freebayes	1.3.1-17- gaa2ace8	( Garrison & Marth, 2012)
Hifiasm	0.15.3-r339)	( Cheng *et al.*, 2021)
HiGlass	1.11.6	( Kerpedjiev *et al.*, 2018)
Long Ranger ALIGN	2.2.2	https://support.10xgenomics.com/genome-exome/ software/pipelines/latest/advanced/other-pipelines
MitoHiFi	2.0	( Uliano-Silva *et al.*, 2021)
PretextView	0.2	( Harry, 2022)
purge_dups	1.2.3	( Guan *et al.*, 2020)
SALSA	2.2	( Ghurye *et al.*, 2019)

### Ethics/compliance issues

The materials that have contributed to this genome note have been supplied by a Darwin Tree of Life Partner. The submission of materials by a Darwin Tree of Life Partner is subject to the Darwin Tree of Life Project Sampling Code of Practice. By agreeing with and signing up to the Sampling Code of Practice, the Darwin Tree of Life Partner agrees they will meet the legal and ethical requirements and standards set out within this document in respect of all samples acquired for, and supplied to, the Darwin Tree of Life Project. Each transfer of samples is further undertaken according to a Research Collaboration Agreement or Material Transfer Agreement entered into by the Darwin Tree of Life Partner, Genome Research Limited (operating as the Wellcome Sanger Institute), and in some circumstances other Darwin Tree of Life collaborators.

## Data Availability

European Nucleotide Archive: *Meganola albula* (Kent black arches). Accession number PRJEB46332; https://identifiers.org/ena.embl/PRJEB46332 ( Wellcome Sanger Institute, 2022). The genome sequence is released openly for reuse. The *Meganola albula* genome sequencing initiative is part of the Darwin Tree of Life (DToL) project. All raw sequence data and the assembly have been deposited in INSDC databases. The genome will be annotated using available RNA-Seq data and presented through the Ensembl pipeline at the European Bioinformatics Institute. Raw data and assembly accession identifiers are reported in Table 1.

## Author information

Members of the University of Oxford and Wytham Woods Genome Acquisition Lab are listed here: https://doi.org/10.5281/zenodo.4789928.

Members of the Natural History Museum Genome Acquisition Lab are listed here: https://doi.org/10.5281/zenodo.4790042.

Members of the Darwin Tree of Life Barcoding collective are listed here: https://doi.org/10.5281/zenodo.4893703.

Members of the Wellcome Sanger Institute Tree of Life programme are listed here: https://doi.org/10.5281/zenodo.4783585.

Members of Wellcome Sanger Institute Scientific Operations: DNA Pipelines collective are listed here: https://doi.org/10.5281/zenodo.4790455.

Members of the Tree of Life Core Informatics collective are listed here: https://doi.org/10.5281/zenodo.5013541.

Members of the Darwin Tree of Life Consortium are listed here: https://doi.org/10.5281/zenodo.4783558.
