# Supplementary material for: The genome sequence of the Kent black arches, Meganola albula (Denis & Schiffermüller, 1775)
Source: Wellcome Open Res. 2022 Dec 22;7:310. [Version 1] doi: 10.12688/wellcomeopenres.18672.1 (PMC10280029; doi:10.12688/wellcomeopenres.18672.1)
Supplement: Peer review history

# Peer review: Reviewer response for version 1

**DOI:** 10.21956/wellcomeopenres.20707.r59649

**Published:** 2023-06-21

**Authors:** Zheng Li, Allen Xue

**Affiliations:** 1 The University of Texas at Austin, Austin, Texas, USA

In this manuscript, the authors provide a chromosome-level assembly for the Kent black arches moth. The background information was concisely written and the methods for extraction, sequencing, and assembly were easy to follow and understand. The data and figures were well-organized, making the findings clear and accessible.

 However, the authors should clarify how they identified or confirmed which scaffold was the Z chromosome. They should also indicate what the sex determination system for this species is (ZW or ZO), if known.

 The sample acquisition portion of the methods section could use additional information concerning how the specimens were identified and distinguished from other moth species. 

 Minor points

- ilMegAlbu4 is mentioned as the second specimen, but it should be the third.

Are sufficient details of methods and materials provided to allow replication by others?

Yes

Is the rationale for creating the dataset(s) clearly described?

Yes

Are the datasets clearly presented in a useable and accessible format?

Yes

Are the protocols appropriate and is the work technically sound?

Yes

Reviewer Expertise:

Insect evolution and genomics

We confirm that we have read this submission and believe that we have an appropriate level of expertise to confirm that it is of an acceptable scientific standard.

# Peer review: Reviewer response for version 1

**DOI:** 10.21956/wellcomeopenres.20707.r57471

**Published:** 2023-06-14

**Authors:** Charles Robin

**Affiliations:** 1 The University of Melbourne, Melbourne, Victoria, Australia

This manuscript describes the genome sequencing and assembly of a Lepidopteran species that goes by the quaint common name of ‘Kent black arches’.

 The affiliations imply that the rationale for this project is part of the wonderful Darwin Tree of Life project. Such a rationale is fine by me but I am inferring it from the affiliations and so I have ticked No on the box above.

 I have experience with Lepidopteran genomes and this one seems to stack up well. The length of the assembly and the number of implied chromosomes are consistent with my expectations. The other quality metrics, particularly those in Figure 2, look good too.

 In general, I am disappointed (and don’t really understand why) the heterogametic sex isn’t routinely preferred in genome sequencing projects. We need more W chromosomes! And the consequent ratio of Z to autosomes reads may also help assemblies.

 There is confusion over the first/second/third specimens. The first sentence of the Methods refers to collection of two specimens. But the third sentence refers to ‘ A second specimen’ (isn’t this the third?)

 Figure 3 is too cryptic. What is the Y axis exactly? The key shows grey box but that cannot be identified in the picture. What do the small dots indicate? This is not a self-standing figure.

Are sufficient details of methods and materials provided to allow replication by others?

Yes

Is the rationale for creating the dataset(s) clearly described?

No

Are the datasets clearly presented in a useable and accessible format?

Yes

Are the protocols appropriate and is the work technically sound?

Yes

Reviewer Expertise:

Insect genetics

I confirm that I have read this submission and believe that I have an appropriate level of expertise to confirm that it is of an acceptable scientific standard, however I have significant reservations, as outlined above.

# Peer review: Reviewer response for version 1

**DOI:** 10.21956/wellcomeopenres.20707.r58649

**Published:** 2023-06-12

**Authors:** Tobias Baril

**Affiliations:** 1 Institute of Biology, University of Neuchâtel, Neuchâtel, Canton of Neuchâtel, Switzerland

The authors provide a high-quality genome assembly for the Kent black arches moth, which is available publicly and for reuse immediately. 

 Concise background information on the species is provided, and the introduction is well-written. The sequencing and assembly protocols are clear and well-described, and accessions are provided to access the data generated for this project. Version numbers are present where appropriate for bioinformatics workflows.

 It would be useful for more clarification to be provided on the expert identification of this species to confirm this is indeed the Kent black arches, particularly as the authors state this bares " *close similarity to Scarce Black Arches (Nola aerugula), another species found in similar coastal UK habitats*" and the mentioned scarcity of this species in the UK.

**Minor Points**

- I would suggest changing 'til' to something more appropriate such as "until" or "to" in this context. 
- Adding images of the other specimens used in this study would be a nice touch, and also potentially provide visual support for the identification of the species. 

Are sufficient details of methods and materials provided to allow replication by others?

Yes

Is the rationale for creating the dataset(s) clearly described?

Yes

Are the datasets clearly presented in a useable and accessible format?

Yes

Are the protocols appropriate and is the work technically sound?

Yes

Reviewer Expertise:

Evolutionary Genomics, Bioinformatics tools development, Transposable elements, Comparative Genomics

I confirm that I have read this submission and believe that I have an appropriate level of expertise to confirm that it is of an acceptable scientific standard.

# Peer review: Reviewer response for version 1

**DOI:** 10.21956/wellcomeopenres.20707.r56894

**Published:** 2023-06-05

**Authors:** Raghuvir Viswanatha

**Affiliations:** 1 Department of Genetics, Blavatnik Institute - Harvard Medical School, Boston, Massachusetts, USA

The authors provide an accessible, high-quality genome for the Kent black arches, a species of moth.

 The authors should provide more detail about the processes used by the experts to authenticate the species. In particular, they should outline the observations they used to avoid misidentification.

 Based on comparison of the genome sequence of ilMegAlbu1 to the RNA sequence of ilMegAlbu4, the authors should state their confidence that these samples are of the same species.

 Minor points

- At least one instance of ‘till’ should be changed to ‘until’.
- The authors might present images of the other specimens used in the study (ilMegAlbu2 and ilMegAlbu4).
- The authors might make a statement about how improved coverage could be obtained and how chromosome phasing could be achieved.
- The authors might include a sentence introducing outstanding biological questions that could be addressed with the data presented (e.g., to understand the mechanism of recent changes in species abundance, etc…)

Are sufficient details of methods and materials provided to allow replication by others?

Yes

Is the rationale for creating the dataset(s) clearly described?

Yes

Are the datasets clearly presented in a useable and accessible format?

Yes

Are the protocols appropriate and is the work technically sound?

Yes

Reviewer Expertise:

Genetics and functional genomics. I cannot assess the details of genome assembly methods.

I confirm that I have read this submission and believe that I have an appropriate level of expertise to confirm that it is of an acceptable scientific standard.
